# Mammography screening: views from women and primary care physicians in Crete

**DOI:** 10.1186/1472-6874-8-20

**Published:** 2008-11-07

**Authors:** Maria Trigoni, Frances Griffiths, Dimitris Tsiftsis, Eugenios Koumantakis, Eileen Green, Christos Lionis

**Affiliations:** 1 University of Crete, Head of Department of Social Work, University Hospital of Heraklion, Crete, Greece; 2 Health Sciences Research Institute, Warwick Medical School, University of Warwick, UK; 3 Head of Department of Surgical Oncology School of Medicine, University of Crete, Greece; 4 Head of Department of Obstetrics and Gynecology School of Medicine, University of Crete, Greece; 5 Director of Centre for Social and Policy Research, University of Teesside, UK; 6 Head of Clinic of Social and Family Medicine, School of Medicine, University of Crete, Greece

## Abstract

**Background:**

Breast cancer is the most commonly diagnosed cancer among women and a leading cause of death from cancer in women in Europe. Although breast cancer incidence is on the rise worldwide, breast cancer mortality over the past 25 years has been stable or decreasing in some countries and a fall in breast cancer mortality rates in most European countries in the 1990s was reported by several studies, in contrast, in Greece have not reported these favourable trends. In Greece, the age-standardised incidence and mortality rate for breast cancer per 100.000 in 2006 was 81,8 and 21,7 and although it is lower than most other countries in Europe, the fall in breast cancer mortality that observed has not been as great as in other European countries. There is no national strategy for screening in this country. This study reports on the use of mammography among middle-aged women in rural Crete and investigates barriers to mammography screening encountered by women and their primary care physicians.

**Methods:**

Design: Semi-structured individual interviews. Setting and participants: Thirty women between 45–65 years of age, with a mean age of 54,6 years, and standard deviation 6,8 from rural areas of Crete and 28 qualified primary care physicians, with a mean age of 44,7 years and standard deviation 7,0 serving this rural population. Main outcome measure: Qualitative thematic analysis.

**Results:**

Most women identified several reasons for not using mammography. These included poor knowledge of the benefits and indications for mammography screening, fear of pain during the procedure, fear of a serious diagnosis, embarrassment, stress while anticipating the results, cost and lack of physician recommendation. Physicians identified difficulties in scheduling an appointment as one reason women did not use mammography and both women and physicians identified distance from the screening site, transportation problems and the absence of symptoms as reasons for non-use.

**Conclusion:**

Women are inhibited from participating in mammography screening in rural Crete. The provision of more accessible screening services may improve this. However physician recommendation is important in overcoming women's inhibitions. Primary care physicians serving rural areas need to be aware of barriers preventing women from attending mammography screening and provide women with information and advice in a sensitive way so women can make informed decisions regarding breast caner screening.

## Background

Among women, breast cancer is the most commonly diagnosed cancer both in the developed and developing world and a serious cause of mortality and morbidity [1-4]. There is evidence from many countries that breast screening with mammography can reduce mortality from breast cancer [5-9] and mammography screening has been recommended in Europe for over a decade [10]. In 2006 in Greece, the age-standardised incidence and mortality rate (ASRs, European Standard) for breast cancer per 100.000 was 81,8 and 21,7 [11]. Although this is lower than most other countries in Europe, the fall in breast cancer mortality observed in most European countries over the last decade has not been as great in Greece [12]. During the 1990s, the observed incidence of female breast cancers increased in Europe, accompanied by a significant decrease in breast cancer mortality [4]. Many European countries, including the Scandinavian countries, Germany, Poland, the Czech Republic, Austria, Switzerland, Italy and Spain have shown an appreciable reduction in mortality rates (between 8% and 19% in the last 5 years), which has been attributed to earlier detection and improved treatment [11]. Reductions in mortality rates have been lower in Greece [11], where delayed diagnosis seems to be a key issue. There is no nationally formulated strategy for early detection of breast cancer, and mammography screening programmes have yet to be established in the Greek mixed public-private health care system. There have been local initiatives such as a pilot study by the Hellenic Society of Oncology [13] for early detection af breast cancer. In this pilot study in Ilia and Messinia in the Peloponesos, in Southern Greece women aged 40–64 years were invited for screening and a participation rate of 52,48% was reported [13]. The establishment a mobile mammography unit to cover rural population health needs has been proposed and is currently being set up. Although there is free access for all to health care services through the social insurance system [14], use of the private sector, including private diagnostic centres, is on the increase [15] but it is mostly people with higher education and income levels that use these centres [15]. Where mammography screening has been promoted, for example through primary care, participation rates have been low [16]. Debate on health care reform in contemporary Greece is focused on primary care enhancement and health promotion, including the encouragement of appropriate mammography breast screening [14]. However, Greek general practitioners report that heavy workloads and lack of time make it difficult for them to engage in prevention and health promotion activities [17]. Little is known about how women in Greece perceive mammography breast screening. This study explores the knowledge, attitudes and perceived practices of both primary care physicians and women in relation to mammography breast screening on the island of Crete in Greece. The study was led by a research team in Crete, building on earlier work in the UK [18]. It forms part of a wider programme of research undertaken in Crete to identify the key components of a regional policy for breast cancer screening.

### Mammography screening participation in differing health care systems

Studies of mammography screening participation rates and reasons for participation or non-participation have been undertaken in many countries with diverse health care systems and screening programmes. In the UK, where there is a well-established population-based screening programme with invitations sent to eligible women every three years, women find mammography screening an uncomfortable experience, but they perceive attendance as a social obligation [18]. No such scheme exists in Greece, where women must seek advice and care on their own initiative. This paper therefore briefly reviews only studies undertaken with women in health care systems similar to the Greek mixed public-private model. Similarly, it reviews studies of physicians' attitudes to mammography screening undertaken in countries with a mixed health economy.

In Europe, studies in France [19] and Spain [20] show participation to be higher among women of higher income and higher educational attainment. In North America, similar trends are found both in the USA [21] and Canada [22]. Where studies have asked women why they participate or not, a range of reasons has been found. For example, reasons given for non-participation by Spanish women included fear of finding a serious problem and the difficulty of making and keeping an appointment [20]. In the US, non-participating women perceived the test to be unnecessary in the absence of symptoms and believed that they were not themselves at risk of cancer. Other concerns included inconvenience, discomfort, embarrassment and pain [21]. A study with non-participating Canadian women identified similar issues, with the addition of rurality reducing participation. In the US, rural women were less likely to receive mammography screening at recommended intervals [23]. Several studies indicate that older women may be unaware that they run a greater risk of developing breast cancer than younger women. Furthermore, it would appear that they perceive mammography to be unnecessary in the absence of symptoms [21,24]. Older women have been found to be more negative about the outcome of cancer; their failure to attend screening is related to knowledge and information barriers [25]. As a result, they undergo fewer early-detection examinations than younger women [26].

In countries such as the UK, a woman's personal physician is not involved in arranging mammography screening. However, studies have shown that in countries with a mixed health economy, recommendation by a physician is one of the most powerful incentives for women to attend mammography [27,28] regardless of age, socioeconomic status or ethnic group [26,29]. A study in Cyprus found that physician recommendation and women's sense of self-effectiveness were the most important predictors for the decision to undergo screening [30]. Studies with physicians report difficulties concerning implementation of preventive care; the most important barriers reported were lack of time [17,31,32], lack of patient compliance with advice [31], heavy workload [17,32] and no reimbursement [17]. Conflicting professional recommendations for screening older women, leaving older women out of clinical trials of screening efficacy, and possible negative attitudes held by physicians and patients all contribute to lower screening rates among older women [29]. Physicians' practices and attitudes in recommending screening vary according to age, years of training, speciality and gender [33]. Some studies have also demonstrated a higher rate of referral among women physicians [3,34,35].

The aims and design of our study were underpinned by a model of transcultural health care utilization [36] previously tested in rural and urban Crete, where biomedical and indigenous knowledge systems co-exist [37]. This model identifies a series of factors that interact with utilization to varying degrees. On the individual level, the model includes predisposing factors such as socio-demographic characteristics (age, education, work status, marital status), psycho-social characteristics (attitudes towards health care, knowledge and practices) and enabling factors (income of household, socio-economic status, financial cost). These all influence the possibility of using health care at the individual level. On the medical system level, factors such as geographical and financial accessibility affect the influence of the medical system on the choice of type of health care. Our study aimed to determine what influences the uptake of mammography screening in rural Crete at both the individual and medical system level. The model guided the selection of questions for the interviews with both physicians and women.

Our research questions were as follows

a) What attitudes do middle-aged women in Crete have towards the use of mammography screening, and what do they know about it?

b) What factors influence the women to attend mammography screening?

c) What are the views of physicians in Crete concerning women's participation in mammography screening?

d) Do physicians follow guidelines on mammography screening when they advise women?

The study focused on women in rural Crete, and explored the perspectives of physicians working in publicly funded rural health centres on the island, since it was undertaken to assist in the development of a regional policy for breast screening in Crete. As the aim was to explore the approach women and physicians take to mammography screening, data was collected by means of qualitative interviews [38].

## Methods

### Setting

The 14 Primary Health Care Centers (PHCCs), serving the rural population of Crete (283,694 residents), were included in this study. Primary Health Care Centres are staffed by GPs, internists (total number of doctors = 105), nurses, midwives, health visitors, lab assistants, and other administrative personnel, and provide health promotion, prevention and acute care services free of charge for all who attend the centres [14]. Agreement to participate in the study was sought from the director of each PHCC.

### Participants

Thirty women attending the PHCCs during the study period (March-June 2004) were recruited. In order to obtain a broad range of views and experiences, we aimed to recruit a random sample closely representing all the different rural areas in Crete. The sample was drawn from the list of regular appointments at every Health Centre. The interviewer (MT) attended each PHCC on a set day and approached the first two women to attend, provided they were residents of the catchment area covered by the centre, aged 45–65 years, and were attending for a regular check-up appointment with a GP or internist. Every woman approached was interested and agreed to participate, and after the interview many women asked for more information about mammography. Twenty-eight primary care physicians (PCPs) were recruited. The interviewer asked the two physicians on the morning shift for an interview. Where more than two were working, two were selected at random. One physician refused, so a physician working the next shift was asked to participate. The study recruited physicians only, as they are the PHCC health professionals who give individual women advice about mammography screening. Participants received written information about the study's aim, the voluntary nature of participation and assurance of confidentiality. All were asked to sign a consent form. Interviews took place in the primary care centre and lasted 30–45 minutes.

### Interview development

Semi-structured interview schedules consisting of open questions were used. The interview schedules were a translated and adapted version of those previously used in the UK [39], one for physicians (see additional file 1) and one for women (see additional file 2). The women's interview schedule covered the women's social relationships; their priorities and concerns about their health; knowledge and attitudes to mammography screening and about its safety; their experiences with health professionals and the decision making process in relation to mammography screening. Care was taken to avoid making suggestions to women about their reasons for use or non-use of mammography screening. The health professionals' interview schedule covered physicians' perspectives on health priorities and concerns facing women in mid life, their views on women's health in mid life, their knowledge and attitudes to mammography screening and how they approach the decision making process about mammography with women. This paper focuses on the data concerning mammography screening. Data collected on wider social and health issues provide an understanding of the context for women and health professionals, which aids interpretation of the data.

### Analysis

All interviews were audio taped and transcribed by the principal investigator. The transcripts were read by CL who contributed to the thematic analysis. Analysis of the interview data was undertaken through a process of close reading of the data, identifying key themes, relating the themes to relevant literature in the field and then returning to the data [40,41]. All the interviews, the coding and initial analysis was undertaken in Greek. The initial analysis report, including relevant quotations from the data, was translated into English and the results of the analysis were discussed among the whole research team. From this discussion, further analysis of the Greek data was undertaken and a final analysis developed in English.

### Ethics

The Scientific and Ethics Committee of the University Hospital of Crete approved the study.

## Results

This paper describes the 30 women and 28 physicians interviewed and then reports the thematic analysis, first of the women's interviews and then the interviews with the physicians.

### Participants characteristics

Of the 30 women who participated in the interviews (mean age of 54,6 years; SD 6,8), 15 women had undergone mammography (ages: 45–50 years n = 6; 51–55 years n = 3; 56 – 60 years n = 4; 61 to 65 years n = 2) and others 15 had never had mammography (ages: 45–50 years n = 5; 51 to 55 years n = 2; 56 – 60 years n = 2; 61 to 65 years n = 6). Table 1 gives the socio-demographic characteristics of the women interviewees and use of mammography as reported during the interviews. Most of the women had low income and limited final education levels. The reported use of mammography does not distinguish between mammography screening and mammography undertaken as part of a process of diagnosis of a breast abnormality.

**Table 1:** Women interviewees' reported socio-demography and use of mammography

**Age group (years)**	**Number of women**	**Number of women who have used mammography**	**Number of women who have never used mammography**
45–50	11	6	5
51–55	5	3	2
56–60	6	4	2
61–65	8	2	6
**Marital status**			
Widow	3	2	1
Married	26	13	13
Single	1		1
**Education completed**			
No schooling	1	1	
Pimary school	21	8	13
Secondary school	5	3	2
High school	1	1	
Higher education	2	2	
**Work status**			
Retired	4	1	3
Private employee	9	5	4
Domestic/agricultural work	11	6	5
Full time house wife	5	2	3
Public office	1	1	
**Weekly household income before income tax (euro)**			
0–100	7	3	4
100–200	12	6	6
200–300	7	3	4
300–400	1	1	
400–500	1	1	
500–700	1		1
Up 700	1	1	
**Reported use of mammography**			
Never	15		
Every year	4		
Once	6		
Two – three times	5		

Of the 28 physicians interviewed (mean age 44,7 years; SD 7,0), 13 were male and 15 female. Six physicians were qualified as internists and 22 as general practitioners. Table 2 summarises their age group, length of time working as a physician and length of time in their current post. More than half of the physicians had worked in their current post for less than ten years.

**Table 2:** Physicians' age, years working as a physician and years in current post

**Characteristic**	**Number**
***Age***	
*30–35*	4
*36–40*	4
*41–45*	3
*46–50*	13
*51–55*	2
*56–60*	1
***Gender***	
*Male*	13
*Female*	15
***Specialty***	
*Internal Medicine*	6
*General Practitioner*	22
***Total years of work***	
*6–10*	12
*11–15*	3
*16–20*	6
*21–25*	6
*26–30*	1
***Total years of work in current position***	
*0–5*	12
*6–10*	4
*11–15*	7
*16–20*	4
*21–25*	1

### Mammography screening from the women's perspective

#### Women's knowledge of mammography

Most of the women seemed to be aware of mammography and had a general idea of what it was. Of the 30 women interviewed, over half (18) knew that mammography was an examination of the breast and a further 7 knew that it was a preventive examination for breast cancer.

It prevents breast cancer and...... what else now? It is a preventive check for the breast. I tell other women about mammography, when we talk about it, I tell them to go do mammography, there is an easy, let's say, solution for the breast......... if there is a problem (woman 19).

Mammography is a test you do for prevention of breast cancer. It takes place once a year, and clinical breast examination once a year. One time, as I have done, one time we do mammography and every six months clinical breast examination and observation, let's say, if something happens in this duration. (woman 21).

Five women knew nothing about mammography screening (aged 48 – 65 years). One woman (age 52 years) talked about breast self-examination but did not know about mammography screening.

The women interviewed had learned about breast cancer, breast self-examination and mammography from various sources including health professionals (n = 17), mass media sources (n = 12), family or friends or when they heard about a new case of breast cancer. (n = 11). The majority of women (n = 23) said they trusted the physician's expertise on medical issues such as mammography.

God has appointed doctors to save and help people and if you meet a good doctor he will do good work (woman 15)

In the beginning, I ask first of all the doctor. Yes, ....yes the doctor as an expert, I must ask for him to inform me. I will do what he will say to me because he is the expert, he knows better. (woman 22).

This may suggest that doctors enjoy high status and are revered by some women in Crete.

#### Women's use of mammography

Of the 15 women interviewed who had never had a mammography for any reason (screening or diagnostic), 6 said no one had recommended mammography screening, but if it had been recommended, they would have agreed to it. A further three of the 15 women said no one had ever informed them about it, leading them to assume that they didn't have any particular need for it. This may, at least in part, reflect the trust women place in their doctor as the revered expert.

Of the 15 women who had undergone mammography, six women reported having a mammography test because their gynaecologist or endocrinologist (private physician) suggested it, two were recommended mammography by a physician at a PHCC, one by a midwife, and six women said they decided to undergo mammography themselves or after discussion with their daughter or friend. It is unclear, particularly for those women having mammography once or two to three times, when the mammography was for screening purposes or when it was because of a problem with their breast. Generally these women were unclear about what mammography screening could achieve. This confusion might be related to a lack of engagement with health promotion literature on the subject and the impact of cultural attitudes that suggest that absence of symptoms indicates good health.

### Barriers to mammography screening

#### Absence of symptoms

This section reports what women said about mammography screening, particularly what put them off going for screening. Although asked about screening, nine women specifically cited absence of symptoms as a reason not to have mammography, of whom one had already experienced mammography and eight had not.

These findings and the following women's responses suggest that the benefits of screening programmes are either poorly understood or that screening is rejected as premature intervention.

I've no breast problems, so mammography isn't necessary. (woman 15)

We aren't the type of people who go to the doctor if we only have a pain or some health problem. If we see some trouble, we go to the doctor, but then it's a little late (laugh), a little late.(woman 4).

Knock on wood, I don't know because I don't have that problem. (woman 15)

We must be checked, we must be examined, but you must have some problem, eh? Without a problem should you go? No, I have never gone to a doctor about that.(woman 11)

It is unclear from the interviews whether these responses reflect a lack of understanding of screening for early detection and treatment of breast conditions, or whether this is understood but the women do not welcome such health interventions, viewing them as unnecessary.

#### Risk and Safety Issues

When asked about the safety and risks of mammography screening, seven women mentioned that fear of the effect of exposure to radiation put them off. Six of these women had experienced mammography.

The radiation that I'll be exposed to. I also think about that because I have been having many examinations lately, but... if it is necessary. I think that we are getting radioactivity now but how harmful it is to our health I don't know. (woman 19)

Six women said they had not had mammography screening because a physician had not recommended it, displaying a trust in physicians to recommend screening if they needed it. None of this group had experience of mammography either and they reported both no encouragement or recommendation for breast screening by their doctor, and a lack of funds to pay for the screening itself. However, half of this group said they would have a mammogram if their physicians recommended it

I didn't ask the doctor. And since I didn't ask they haven't mentioned it. If a doctor says that I have to have a mammogram, yes, I will have it. (woman 24).

It depends on what information the doctor will give to me to continue if it is necessary to do it When he says that it is must to do the mammography I will do it. If I see something to my breast and I will visit the doctor and he says to me that you must do a mammography, because the doctor says it and he knows better I will do it. (woman 13).

Six women said they knew they should go and have mammography screening but they had not got around to doing so. Five of the six women have never undergone mammography.

#### Fear and Negativity towards Mammography

Fear of pain or a previous traumatic experience emerged as another important reason for not attending mammography screening for five of the women.

I don't know. Sometimes as my breast is compressed, perhaps they'll crush something. (woman 18)

Four women talked of their fear of finding something seriously wrong such as cancer. Two of the four had experience of mammography.

Because we are afraid, that maybe, let's say, you have something in your breast and you will need various other procedures and for this reason, you avoid being checked. (woman 24)

One woman had been frightened by the experience of her neighbour who had a mammogram and later on lost her breast so had not had a mammogram herself.

Yes I am afraid of the expected results. (woman 10)

#### Not a Priority

Four women mentioned they were too busy with family obligations to attend mammography screening. Four women said embarrassment put them off attending:

If it is something gynaecological, I may be embarrassed because here in the countryside we are, let's say, more... um... but since I've never needed to have a breast examination, a doctor to examine my breast, I don't know.(woman 7).

Other reasons that women mentioned included the cost of having a mammogram (n = 2), the lack of information about having a mammogram (n = 2), the lack of free time (n = 2), the difficulty of getting to the mammography centre due to the distance (n = 1):

Nah. It's mostly the distance for me (woman 20)

### Mammography screening from the physicians' perspective

#### Knowledge, attitudes and practices

Most physicians in the sample were well informed. When asked about their knowledge of screening for breast cancer with mammography, the majority (n = 20) claimed to be well-informed although seven said they would like more information. However, one physicians said:

It isn't my specialty; I can't say that I am informed. (physician 26)

Another physician expressed doubts about the reliability of mammography screening and whether it should be trusted. He said:

I'm not well informed about whether it gives reliable information. (physician 12)

The majority of physicians interviewed talked about the importance of their recommendation for women to attend mammography screening (n = 16), confirming that when they urge women to undergo mammography screening most of them will do so.

If you tell them that the mammogram is necessary, all of them have it. (physician 22)

Just one physician had doubts and felt unsure of how to persuade women of the importance of mammography screening and did not feel prepared to manage a large number of patients:

According to the guidelines I feel that I do relatively well. As far as management of large numbers of patients, such as those who come to the office, I don't feel prepared. I don't consider myself well-informed on this subject, neither about how I will present it to the patient, nor how I will persuade her of the necessity of this examination." (physician 3)

#### Lack of time for adequate discussion in the clinic

Another notable finding was that a number of physicians (5) reported lack of sufficient time to discuss mammography screening because of the large number of patients attending each clinic and the short time available to talk to each one.

When the patient visits (the clinic) at regular office hours we will almost always propose that a mammogram be done. However, when the visit occurs during the emergency shifts we often neglect it. (physician 3)

#### When is mammography screening recommended by physicians?

When asked for whom they would recommend mammography screening, physicians mentioned

- women in the appropriate age range to be screened (n = 9)

- women who may have a hereditary predisposition towards breast cancer (n = 12)

- women who asked for a mammography of their own accord (n = 2)

In total, only eight physicians reported suggesting mammography to all eligible women. Although interviews were about mammography screening, eleven physicians mentioned they would recommend mammography to women presenting with clinical symptoms or who had palpable nodules (n = 11).

If there is a hereditary case-history then I insist more, or of course if there is a clinical finding, in the breast examination. (physician 5)

The age, a possible case-history and hereditary predisposition. (physician 19)

During interviews physicians were quite clear about the subtle difference between screening and diagnostic mammography, screening being for asymptomatic women and diagnosis for women with concerns about disease based on symptoms and signs. However, in the health care setting the actual referral procedure for mammography screening was the same for both screening and diagnosis, which may be one reason why the majority of women in our sample were confused about the purpose and benefits of mammography screening.

#### Barriers to Mammography Screening

The primary care physicians interviewed all identified difficulties with arranging mammography for rural patients because of difficulties in scheduling an appointment at public hospitals and waiting times.

They have no access to mammography. The two public hospitals schedule appointments four, five or six months after referral. (physician 2)

MT contacted all seven prefecture general hospitals and the University general hospital of Crete (all publicly funded) to check the availability of mammography and the waiting times for appointments. Only five of the eight hospitals provide a mammography service, so for some PHCC accessing mammography would be very difficult. Those providing mammography claim to have waiting times for an appointment of between two and four months.

#### It's a long way to the clinic for most rural women

Eight physicians reported that distance and transportation (geographical accessibility) difficulties in travelling through mountainous regions to the city were a problem for women, with older women having more transportation difficulties than younger ones:

One woman said to me: Why should I have a mammogram? It's not easy to go to Heraklion. It's too far. (physician 8).

If women were unable to afford the cost of visiting a private diagnostic centre and had to wait for a long period in a public hospital, their physicians usually avoided recommending mammography. Nine physicians mentioned the cost of having mammograms at private diagnostic centers as a barrier, particularly for older patients with fixed, low incomes. One physician said:

The financial side is such a problem that many poor people would rather die than have their lives complicated every year to have a mammogram. I can't talk to them about it. In other words a lot of things stop at the Health Center. If it costs as much as the cost of a trip to Rethymnon they would rather even be diagnosed with cancer to make the trip worth it. (physician 23)

Physicians also identified the absence of symptoms as a factor discouraging women from having mammograms, which is similar to data in the women's interviews:

(Women say) there's nothing the matter with me, why should I have a mammography? (physician 8).

Physicians reported that women were motivated to have a mammogram when a relative has had breast cancer (n = 6) and when the woman had symptoms such as breast pain (n = 12).

Other reasons physicians gave for women not having a mammogram included: not considering it a priority (n = 3); embarrassment (n = 3); fear of diagnosis (n = 4); fear of pain (n = 3) and fear of radiation (n = 8). All of these factors except embarrassment were also mentioned by the women, although a number of physicians identified it as an issue, especially for older women:

Deep down however, most of the time it is embarrassment, especially for older women. Very often it's embarrassment. (physician 6)

Fear, that they will find something,...doctor, I don't want to know (physician 2).

Pain/discomfort and radiation risk were also mentioned as barriers:

Another high percentage considers it a painful exam from their previous experiences. (physician 3)

The main reason is that they will be exposed to radiation. (physician 18)

In addition, family obligations and lack of free time were identified as discouraging women from having a mammogram. One physician said that women tend to say:

I haven't anywhere to leave the kids, it isn't easy to go to Heraklion. I have my father-in-law, my mother-in-law, we don't have time, family obligations (physician 8)

This suggests that women put their own health last, after the well being and care for other family members. Over half of the physicians (n = 16) suggested that physician gender plays a role in determining whether women felt able to discuss sensitive subjects such as their breasts. Two male physicians said they refer women to female physicians or midwives for gynaecological issues. One female physician said that women prefer to consult female physicians as far as prevention is concerned, but when they confront a serious medical problem, they prefer to consult male physicians, which may suggest more deference to the authority of the latter.

#### The importance of patient's characteristics

In response to a question about how educational level, age and socioeconomic status affect women's knowledge and use of mammography screening, the vast majority of physicians (n = 23) agreed that these factors were important. Four physicians specifically commented that educated women made use of mammography more frequently than less educated women and older women were screened less frequently than younger women. However, they thought this was changing as women were becoming generally more informed about health issues. Nine physicians talked about the role of financial difficulties in deterring women in seeking breast screening.

The majority (22 out of 30) of physicians interviewed mentioned that women were influenced in their decisions about mammography screening by their overall attitude towards life; attitude towards life seems to play an important role in determining how they address their health. They claimed that women prioritized their children and other family obligations over their own personal or health problems. In general, there was consensus among physicians in the sample with no indication that their views varied by either age or years spent working as a physician.

## Discussion

This study has identified the subtle interplay of complex factors, from both the women and physicians' perspective, that result in women from rural areas of Crete failing to access mammography screening. We summarise in Table 3 factors that seem to impede the use of mammography in rural Crete. This study delineated that most of the women knew about mammography and were interested in having mammography screening. However, it was also clear that few women were able to take an adequately informed decision about mammography screening and share in decision making about results with their doctor.

**Table 3:** Barriers to mammography identified by women and physicians

**WOMEN**		**PHYSICIANS**
Absence of any symptoms	*Contextual issues*	
Fear of radiation		Access to a mammography screening center
Lack of recommendation of physician		Difficulties in scheduling an appointment in state Hospitals, waiting time
Fear of pain		Distance, transportation problems from mountain regions to the city
Fear of results-diagnosis	*Women related issues*	
Family obligations		Absence of symptoms
Embarrassment		Embarrassment felt by women
Cost		Consequences of radiation
Lack of information		Fear of diagnosis
Lack of free time		Problem of free time and family obligations
Distance from screening centers – Transportation problems		Cost at Private Centres
	*Physician related issues*	
		Physician judgment and management
		Gender of physician

The level of education and the income of people living in rural Crete is low and this was the case for the women in our study. There is evidence that socio-economic factors for example, higher income and higher level of education, are important correlates of use for mammography screening. In a previous French study [19] there is evidence that a high monthly household income or high education level, increased the probability of accessing mammography. The majority of physicians in our study were aware of the impact on screening uptake of low levels of education and income. Both the women and physicians mentioned similar barriers to attending mammography screening, many related to the low socio-economic status of the women.

The reasons cited by both women and physicians as to why a referral for mammography screening is not made, have also been identified in earlier studies [20,21,24,42]. The finding that women fail to prioritise their own health also replicates other research [43]. Previous studies [44-46] have reported that women living in rural areas may be less likely to receive mammography than urban women, and breast cancer screening rates are lower in rural communities. Utilization of preventive health care services is lower in rural populations than in urban populations, possibly as a result of barriers to preventive health care that are characteristic of rural settings (isolated residential settings, lack of transport etc.)[44]. Rural women were found to have the same basic knowledge of breast cancer or perceptions of barriers to mammography, but had more complex attitudes towards breast cancer itself. Access to health care remains an important issue facing many individuals. Barriers to health care include financial factors, socio-economic characteristics of the individuals and the health care delivery system, as well as geographical factors [46]. However, none of the barriers discussed stood out as being more important for the promotion of mammography screening than others, suggesting that a multifaceted approach to the promotion of mammography screening is the most likely to be successful.

This study was undertaken to inform local health care reform in relation to mammography screening. The recruitment rate for participants in the study was very high. Both women and physicians expressed interest in both the study and mammography screening for themselves and for the locality. This interest in the study may have been enhanced by the relative lack of previous qualitative studies in this subject in Greece. As with similar studies elsewhere, the data needs to be interpreted with caution as both women and physicians may have exaggerated their enthusiasm for mammography screening due to the nature and setting of the study. However, the study was successful in enabling negative views to be expressed.

Taken as a whole, the data from both women and physicians suggests that there is a growing understanding of the importance of mammography screening and preventive health care more generally, and that with attention to detail as to how it is promoted and provided, mammography screening could become the norm in Greece as it has become in other European countries. Our findings indicate where such attention to detail needs to be focused.

### Promoting mammography screening among women

Our study suggests that there is still a need for raising awareness in relation to breast cancer and the role of mammography screening, even though most women interviewed had some knowledge of it. The concept of preventive medicine is still largely unknown among primary health care centre populations as demonstrated in a recent European study [17]. Raising awareness in this area includes the need to clarify the difference between mammography screening for early diagnosis and mammography for diagnosis of a perceived breast problem. Fear of cancer, fear of the perceived pain of mammography, fear of radiation, embarrassment and women failing to prioritise their own health are cited by individual women and need attention in Crete; however, these are also issues women mention as barriers in countries with high mammography screening rates [20,21,24].

### Promoting mammography screening and the role of the physician

For some of the women interviewed, the physician was perceived as a key person in recommending mammography screening, although other women had arranged their own mammography screening. To achieve high rates of mammography screening, physicians are likely to be important in promoting a screening programme [17,31,47], particularly initially when women expect endorsement for screening from their physician. Physicians can play an essential role in improving women's participation in screening programs [48] and early detection of cancer [31] through direct recommendations to their patients [4]. There is some evidence to suggest that targeting doctors' involvement in screening is associated with an increase in breast screening attendance [49]. In most previous studies, advice, recommendation or encouragement from health professionals has been found to increase the likelihood of attendance [47]. Over 90% of rural women report that a doctor's recommendation to have breast cancer screening is "important" [4]. The involvement of physicians in establishing a programme requires them to have sufficient time, information and support, including clear guidance on eligibility for screening. The role of the physician in encouraging mammography screening long-term is open to debate, as women may become confident enough to seek screening for them.

### Promoting mammography screening through health care policy and process

The results from this interview study suggest a number of policy and process issues where change could increase the uptake of mammography screening. Having different referral routes could ease the confusion for women between diagnostic mammography and screening mammography. This may also make it easier for physicians to refer all women for screening mammography rather than emphasising diagnosis and screening where there is high risk. The process of obtaining mammography screening needs to enable women to attend relatively easily. Many women mentioned that they need to be able to travel to the screening centre, to afford both the travel and the screening, and to have some flexibility in appointment times. This study underscores the need for continued efforts to provide breast cancer screening to rural communities, including community education interventions and low-cost mobile mammography van services [45]. Attention to these issues will also encourage physicians to recommend screening.

### Study limitations

This was a qualitative study recruiting women attending participating primary care practices. Both physicians and women were asked about screening mammography and both responded, with physicians talking about screening in some detail. It appears that not all of the participating women understood the difference between diagnostic and screening mammography, which may have affected their responses. This was an exploratory study, the results of which will increase our understanding of these issues for health care on Crete. The small size and qualitative nature of the study mean that the results are not generalizable to the whole population. However, there is no strong indication that our sample differed in terms of age, profession, culture and language from other rural Greek populations.

## Conclusion

This study provides valuable insights into women's knowledge, attitudes and use of mammography screening and the knowledge, attitudes and use of mammography screening by primary health care physicians in rural Crete. The study was designed to deliver data through which to inform health policy, prioritizing qualitative data collection, methods and analysis and listening to women's voices. The study's findings build upon previous research in other settings but uniquely, provide health care providers and policy makers in Crete with evidence specific to their locality for the future development of a preventive programme of mammography screening. Barriers to the implementation of a mammography screening programme may be similar across different geographical and national contexts, but demonstrating both the specific factors involved and the distinct local way in which such factors interact, is necessary for the development of robust and appropriate regional policy.

## Abbreviations

PHCCs: Primary Health Care Centers; PCPs: Primary Care Physicians; GP: General Practitioners.

## Competing interests

The authors declare that they have no competing interests.

## Authors' contributions

MT was the principal investigator for the study and undertook data collection, transcribed and analysed the interviews, and wrote the initial manuscript. FG and EG undertook a study visit to primary and secondary health care services in Crete funded by the UK Economic and Social Research Council 'Innovative Health Technology programme of research. FG and EG advised on the study design, discussed initial analysis and contributed to the final manuscript. EK and DT contributed to the study design and gave their comments on the manuscript. CL contributed to each stage of the study development, analysis, reporting, reviewed the analysis and interpretation of data, corrected the first draft, co-designed the contents of the manuscript. CL, MT, FG and EG all made a substantial contribution in preparation of the revised manuscript. All authors have participated in the design of the study and have commented critically on the initial manuscript and have approved the final version of the manuscript.

## Pre-publication history

The pre-publication history for this paper can be accessed here:



## Supplementary Material

Additional file 1 **Health Professional's Interview Schedule** Click here for file

Additional file 2 **Midlife Women Interview Schedule** Click here for file
