# Supplementary material for: Mammography screening: views from women and primary care physicians in Crete
Source: BMC Womens Health. 2008 Nov 7;8:20. doi: 10.1186/1472-6874-8-20 (PMC2588567; doi:10.1186/1472-6874-8-20)
Supplement: Additional file 1 — Health Professional's Interview Schedule [file 1472-6874-8-20-S1.doc]

**1. Health Professional’s Interview Schedule**

1. What would you say are the main health priorities or concerns facing women in midlife?

2. Can you tell me what sort of questions women raise with you in relation to menopause, and has this changed over your working life?

3. Do women mention other people who they have turned to for information about midlife health and wellbeing and if so who? (probe for other health professionals, friends, relatives etc)

4. Can you tell me about your views on women’s health in midlife? For example do you see this as a marked time of ‘change’ or simply a transition?

5.How knowledgeable and up-to-date do you feel about the mammography our study is interested in (screening for breast cancer).

6.Is training on existing and developing mammography for women at midlife available to you?

1. If so, can you tell me what types?
2. Do you take advantage of this?
3. How do you assess their worth

7. Do you use the Internet at all? Do you use it to update your professional knowledge? What about on the subject this study is interested in?

8. Can you tell me how you view this health technology both at a professional level and at a personal level if this applies to you and/or your partner?

9. How knowledgeable are your patients about mammography we are focusing on in this project that is on offer? Are they more familiar with some than other ?

10. What do you think are women's main priorities in regard to decisions about mammography?

11.How do you approach the issue of informing women about the potential risks and benefits of mammography?

1. Do you think this is important?
2. How do women react?
3. What issues do women bring up in relation to risk and benefit?
4. What appears important to women?
5. Is this different from what seems important to you as one of their health professionals?

12. Do most women find the decision to use particular mammography a difficult one? How do you help them with this?

13. How usual is it for you to recommend mammography for women, for example do you wait for women to ask or are you more likely to suggest the mammography directly?

1. If it varies, what type of issue makes you decide to mention it to one woman and not another?

14. Do you consider that midlife women have become the focus of too much medical intervention?

15. Do you have any concerns about addressing the question of the mammography with women?

16. Is the way you approach that discussion affected by women’s individual circumstances?

17. How do you manage the situations where women may disagree with the advice offered?

18. What reasons have your women patients given if they choose not to use the mammography screening?

19.. Do you raise, or do any of your women patients discuss using self-care strategies and or alternative therapies instead of, or alongside, health technologies? Can you tell me what types?

20.Can you tell me how you view such strategies and therapies, and what your professional experiences and knowledge are of them?

1. Can you think about this question in relation to your personal experiences and knowledge?

21.Do you understand these alternative forms of health care as ‘technologies’

22. .Do you think differences such as culture, age, ethnicity, disability and socio-economic status have an impact on women’s knowledge of or attitude towards mammography?

23. Do you think these differences impact on health professional’s relationships with women patients?

24. How comfortable and confident do you feel most women are about voicing their own opinions with you as a health professional?

25 May I ask your age?

26. May I ask if you take advantage of any of mammography? (ask which ones if information not volunteered).

27. How would you describe your ethnicity?

28. Can you tell me your job title and give me a brief description of what that entails?

29. How many years have you been working as a health professional?

30. How many years have you been working in your current post?

31. Do you undertake any professional work in addition to being the practice nurse/GP here seeing patients, eg teaching, research, management, audit, other clinics etc either within or outside the practice?
