# Supplementary material for: Mammography screening: views from women and primary care physicians in Crete
Source: BMC Womens Health. 2008 Nov 7;8:20. doi: 10.1186/1472-6874-8-20 (PMC2588567; doi:10.1186/1472-6874-8-20)
Supplement: Additional file 2 — Midlife Women Interview Schedule [file 1472-6874-8-20-S2.doc]

**2. Midlife Women** **Interview Schedule**

In this study we are interested in women's stories about their middle years. We want to learn how women feel about themselves, their lives, their sense of health and wellbeing and their approach to medical issues and therapies.

**1)**. Can we start, therefore, by your telling me something about yourself and your background, for example your age and whether you live with and share your home with other people?

**2)**. Can you tell me what's happening in your life generally at the moment, for example within your relationships, home, work, leisure time, friendship links.

**3).** How would you describe your own health at the moment?

i) How do you feel at the present time in relation to your body?

**4)**. Have you any concerns for your future health?

i) What do you think will happen to your body in future years?

ii) How do you feel about ageing?

**5)**. What would you say are the main health priorities or concerns facing women in midlife?

**6).**Can you tell me what you know about mammography?

**7)** Can you tell me how you found out about mammography?

**8)** Did you use the Internet at all?

**9)**.Have you any thoughts on the 'safety' or 'risks' of any of mammography?

i) What is your understanding of your results ?

**10)**. You may know women who have had mammography. What impact has their experience had on you?

i) Do you think you have influenced other women that you know, such as friends or family members in regard to mammography?

**11).**Do you consider that midlife women have become the focus of too much medical intervention?

**12)**. Do you, or have you, used any alternative forms of health care?

(Alternative therapies such as aromatherapy, massage, supplements and

lifestyle options such as diet, exercise)

i. Have you used them recently?

ii) Have you used them for the health problem that you are here with today?

iii).Again, as with the question we discussed earlier, do you think your attitude towards alternative forms of health care has influenced other women you know, such as friends or family members?

  iv) Can you tell me how you found out about these alternative forms of health care, and did you use the Internet?

**13)**. Can you tell me about your feelings and experiences of talking to health professionals about your health needs recently. It may help to start with the first health professional you spoke to.

i). Have you found it easy or difficult to talk to any health professionals?

ii)Have you had to develop ways in which to approach sensitive subjects,

such as breast problems, with health professionals?

iii)Have you felt listened to and your views respected, or felt your views

dismissed?

iv)Have the health professionals given information/advice on alternative

forms of health care?

v)Did you feel you received enough information to make a proper

'choice' about your health needs?

vi)Is the sex of the health professional important to you, whether they are male or female?

**14)**. Sometimes choices about health care and treatments have pros and cons, and are not straightforward. Can you tell me how health professionals raised the question of 'safety' and ‘risks’ in relation to health care, such as mammography, with you?

**15)**. Thinking about your relationship with health professionals, can you describe how you think your health care decisions have been taken, for example who takes the decisions/how are decisions arrived at?

i) Did you find health professionals enable you to take the decision or that their judgements take precedence over your choice?

ii) Did you feel you came to a shared decision with the health professional?

iii) Did you feel happy for the health professional to take the decision, as

the 'expert'?

iv) Have you ever received conflicting advice from health professionals on the test mammography we have discussed in the interview and how do you feel about this?

**16)** I have now reached the end of my questions, do you have any questions for me?

I will be listening again to what you have told me and the research team will read a transcript of this interview. Sometimes we find that further questions occur to us that we would like to ask you. Would you be agreeable to me contacting you again? (to ask questions by telephone and/or for a further face to face interview).
